# Research landscape of exosomes in platelets from 2000 to 2022: A bibliometric analysis

**DOI:** 10.3389/fcvm.2022.1054816

**Published:** 2022-12-20

**Authors:** Min Zhan, Shengnan Shi, Xiaoyu Zheng, Wenjie Chen, Linjuan Sun, Yehao Zhang, Jianxun Liu

**Affiliations:** ^1^Xiyuan Hospital, China Academy of Chinese Medical Sciences, Beijing, China; ^2^Graduate School, China Academy of Chinese Medical Sciences, Beijing, China

**Keywords:** platelets, exosomes, CiteSpace, VOSviewer, bibliometrics

## Abstract

**Background:**

Blood-derived exosomes are involved in developing multiple pathological processes, with platelets being the most well-known source. Related studies have become an area of research with significant value and potential. However, no bibliometric studies in this field have yet been identified. We aimed to analyze the hotspots and academic trends of platelet exosome research through bibliometric visualization to actively grasp the research base in this field and track its developmental orientation.

**Methods:**

From 2000 to 2022, we screened all relevant publications on platelet exosome-related research from the Web of Science database, generated knowledge maps using VOSviewer and CiteSpace software, and analyzed research trends in the field.

**Results:**

A total of 722 articles were screened for inclusion based on the search strategy. The number of articles on exosome studies in platelets has expanded vastly. The USA and the People’s Republic of China contributed substantially among 69 countries or regions. Amsterdam University and Semmelweis University are the research institutions with the most publications. The most studied and co-cited journals were the *International Journal of Molecular Sciences* and the *Journal of Extracellular Vesicles*. We identified 4,598 authors, with Nieuwland Rienk having the highest number of articles and Bruno Stefania having the most cited publications. Keywords of great interest include “thrombosis,” “anti-inflammatory,” “anti-apoptosis,” “angiogenesis,” “microparticles,” “miRNAs,” “stem cells,” and “biomarkers,” which are key research areas for future development.

**Conclusion:**

We used bibliometric and visualization methods to identify hotspots and trends in platelet exosome research. Platelet exosome research is widely expanding. Future research will most likely focus on “thrombosis,” “anti-inflammatory,” “anti-apoptosis,” “angiogenesis,” “microparticles,” “miRNAs,” “stem cells,” and “biomarkers.”

## Introduction

Exosomes are nanoscale phospholipid bilayer vesicles of 30–100 nm diameter that are endocytosed and resecreted by cells and contain microRNA, siRNA, proteins, lipids, and other components (1). Exosomes are an extracellular vesicle subtype that releases various cell-specific proteins, lipids, and nucleic acids during cell activation or apoptosis and acts as a mediator of intercellular communication in different physiological and pathological processes (2, 3). In recent years, exosomes have been derived from ceramide and/or endosomal sorting complexes required for transport, and researchers have focused on changes in bioactive substances and possible mechanisms of exosome effects in various physiopathological states. Almost all living cell types (4), including platelets, endothelial cells, and smooth muscle cells, can secrete exosomes to control target cells. Exosome amounts and compositions change in response to different stressors, and cells release different exosomes to participate in physiological and pathological processes in body tissues (5, 6).

Platelets are the primary cells involved in hemostasis and thrombosis and also critical immune cells that interact with target cells *via* various pathways, including direct fusion with the cytoplasmic membrane, cell surface adhesion *via* receptor–ligand interactions, exosomal content release under low pH conditions, and endocytic uptake (7). These complex interactions may directly influence disease progression, with beneficial or detrimental effects on maintaining vascular integrity, namely, inflammation, immune process regulation, and the development of tumors, cardiovascular, and hematological diseases, making platelets promising as biomarkers and therapeutic targets in the pathological stages of various diseases such as infection, inflammation, vascular diseases, and cancer, with important research implications. Platelet-derived exosomes (PLT-Exos) constitute approximately 70% of total serum exosomes and have been shown to have procoagulant and pro-inflammatory activities (8), implicating them in thrombotic complications such as rheumatoid arthritis and atherosclerosis (9).

In 1969, Alan Pritchard coined “bibliometrics” (10). Today, bibliometrics is one of the most popular analytical approaches for investigating a research topic in all its aspects. Bibliometric analysis is a comprehensive knowledge system that uses mathematics and statistics to study the literature’s internal connections and distribution patterns (11). It has been widely used to gain insight into the current state, hotspots, and future research trends in a field and provides researchers with references for choosing research directions (12). Other methods, such as traditional reviews, meta-analyses, or experimental studies, make this impossible. Because of these combined benefits, it has also become increasingly important in assessing research trends and developing guidelines (13).

The number of relevant publications has increased dramatically as the study of platelet exosome mechanisms has progressed. Several traditional reviews (14–16) have described the application and role of exosomes in platelets. However, to our knowledge, there are no bibliometric studies on exosome research in the platelet field. We aimed to present the current status and emerging trends in research on exosomes in platelets using a validated bibliometric analysis to provide researchers in the field with qualitative, semi-qualitative, and chronological aspects to identify, evaluate, and visualize the global work on exosomes in platelet research.

## Materials and methods

### Data collection strategy

**FIGURE 1:**
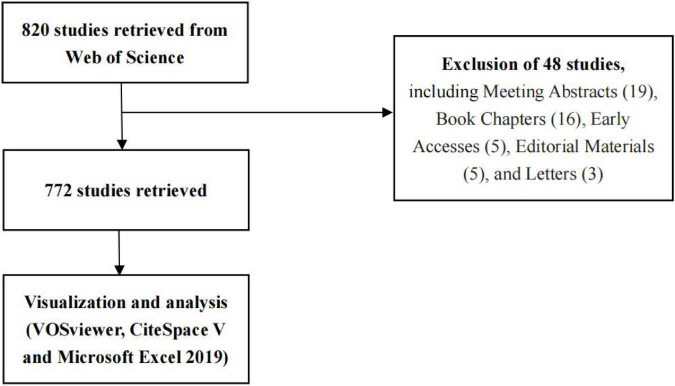
Literature screening for exosome studies in platelet.

The Web of Science (WoS), an authoritative database widely recognized by researchers worldwide, covers a comprehensive range of fields and is widely used in scientific research. In this study, we used the WoS Core Collection to search for exosomes in platelets from 2000 to 2022. The following were the search terms: TS = “Exosom*” AND “platelet*” AND Language = (English) AND with 820 records, Publication Date = (2000-01-01–2022-07-16). After eliminating invalid documents, such as meeting abstracts, editorial materials, early accesses, book chapters, and letters, we finally included 772 records. All search results were downloaded in “full record and cited references,” “record content,” and “plain text” file formats using the download_txt format name. Figure 1 depicts the screening process for relevant research literature. The 772 articles retrieved in this study were cited 42,893 times, with an average of 55.56 citations per article and a high H-index of 106 (Table 1). Many highly cited articles on exosomes in platelet research have been published, highlighting the depth of research and quality content. All searches were conducted on 2 August 2022 to avoid the bias associated with database upgrades. In addition, this study used publicly available databases, so an ethics declaration was not needed.

**TABLE 1:** Bibliographic statistics of 772 recorded publications extracted from WoS.

Total publications	H-index	Sum of times cited	Citing articles	Average citations per item
772	106	42,893	24,918	55.56

### The bibliometric analysis method

In bibliometric analysis, visualization provides a comprehensive and multifaceted view of collaborative networks, research hotspots, and trends in emerging research areas. In this study, we imported all WoS-based search records into Microsoft Excel 2019 and performed the visual analysis with VOSviewer and CiteSpace software.

Microsoft Office Excel 2019 was used to manage the database and analyze the global annual number of publications related to exosomes in platelets and the number of citations to demonstrate the global trends in the field.

CiteSpace is a Java-based citation visualization and analysis software based on scientometrics and data visualization (17). It is adept at exploring collaborations, key points, internal structures, potential trends, and field dynamics, visualizing research hotspots and evolutionary processes, and predicting trends in each field (18). CiteSpace 6.1.R1 was used in this study to generate maps of network visualization (countries/regions, institutions, references), clustering analysis and timeline visualization (keywords), and citation bursts (references and keywords), including a dual map of journals to explore changes in research directions and trends by mining key research themes, outstanding researchers, popular journals, and the institutions and countries with the highest contributions.

VOSviewer is a scientometric tool that provides visual presentations for web-based data creation and is currently used in many fields (19, 20). It provides a full range of visual analysis maps of the literature in different fields and connects *via* co-citation links, co-occurrence, citation, and bibliographic coupling. VOSviewer software offers three types of visual maps: network, overlay, and density visualization (21). In this study, VOSviewer was used to generate maps for network visualization (journals, authors, keywords), overlay visualization (countries/regions, institutions, authors, keywords), and density visualization (countries/regions, institutions, journals, keywords), which along with CiteSpace visualization maps reflect collaborative relationships and hot academic trends.

On 6 August 2022, we obtained the 2021 Impact Factor (IF) and Journal Citation Report division of journals from the Web of Science InCites Journal Citation Report. SCImago Journal and Country Rankings^1^ and eigenfactor^2^ websites were used to obtain the H-index and eigenfactor scores, respectively.

## Results

### Publishing trends and global contributions

**FIGURE 2:**
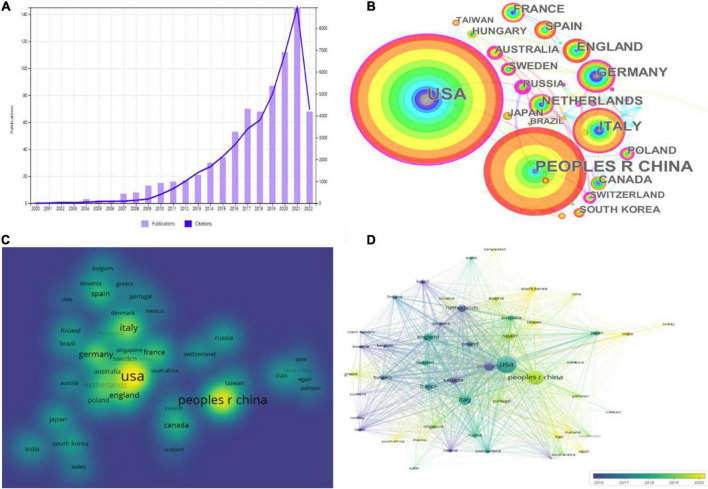
Platelet exosomes in global publications. **(A)** Annual publications and citations of platelet-related exosome studies worldwide. **(B)** CiteSpace-based web visualization of article distribution by country. **(C)** VOSviewer-based density map distribution of articles by country. **(D)** VOSviewer-based chronological order of countries.

After developing the search strategy in conjunction with the study objectives and limiting the type of literature and language, we finally included 772 articles about platelet exosomes. Analysis of the annual number of publications and frequency of citations (Figure 2A) revealed the trend of a significant increase in the number and frequency of citations of studies on exosomes in platelets from year to year, which remained largely synchronized (the number of publications and frequency of citations decreased due to the cutoff date of 2 August 2022 for the search of the literature), notably, after sluggish growth from 2014 to 2017 (30–70 publications per year, 1,669–3,395 citations per year) and explosive growth from 2018 to 2021 (68–145 publications per year, 3,803–8,941 citations per year). The continued popularity of exosomes in platelet-related research indicates widespread interest in this field of application worldwide.

In total, 772 articles were published in 69 countries/regions worldwide, and Table 2 shows the top 10 countries/regions with the highest publication contributions. CiteSpace and VOSviewer were used to create a network visualization of the distribution of countries/regions where global publications are located, with CiteSpace displaying 69 nodes and 92 connections with a network density of 0.0392 (Figure 2B); VOSviewer set the threshold value of the minimum number of publications in a country to two, and there were 69 countries, of which 51 reached the threshold value (Figures 2C,D).

**TABLE 2:** The top 10 countries that contributed publications on exosomes in platelets.

No.	Country	Publications	Centrality	Citations	Average citation rate
1	USA	214 (27.72%)	0.28	11,551	53.96
2	People’s Republic of China	148 (19.17%)	0.05	4,383	29.61
3	Italy	77 (9.97%)	0.12	5,847	75.94
4	Germany	58 (7.51%)	0.24	4,338	74.79
5	England	49 (6.35%)	0.00	3,454	70.49
6	Netherlands	43 (5.57%)	0.19	5,004	116.37
7	France	36 (4.66%)	0.18	2,533	70.36
8	Canada	35 (4.53%)	0.07	715	20.43
9	Spain	35 (4.53%)	0.07	4,194	119.83
10	Poland	25 (3.24%)	0.22	1,747	69.88

Looking at the global distribution of research publications on exosomes in platelets, the USA (214, 27.72%) and the People’s Republic of China (PRC) (148, 19.17%) are the most productive countries, followed by Italy (77, 9.97%) and Germany (58, 7.51%). The top two countries accounted for roughly half of all publications on exosomes in platelet-related research. Several countries/regions, including the USA (0.28), Germany (0.24), and Poland (0.22), demonstrated high centrality among them, as shown by the rosy red circles in Figure 2A, implying that these countries may play a significant role in the study of exosomes in platelets. Then, density visualization plots were used to better visualize all publication centers (Figure 2C), and overlay visualization could represent the year of publication concentration in each country/region. The top three countries in terms of total citations were the USA (11,551 citations), Italy (5,847 citations), and the Netherlands (5,004 citations). In terms of total link strength, the USA (177,267), the PRC (88,783), and Italy (80,066) are the most prominent, indicating that the USA and PRC have the dominant influence in this field. When combined with the year of concentration of country/region publications (Figure 2D), the publications from the USA, Italy, United Kingom, and France were primarily concentrated in 2016–2019, while those from the PRC, Spain, India, and Iran were primarily concentrated in 2019–2022. The USA, Germany, Poland, and other countries were actively cooperating.

### Institutional distribution analysis

**FIGURE 3:**
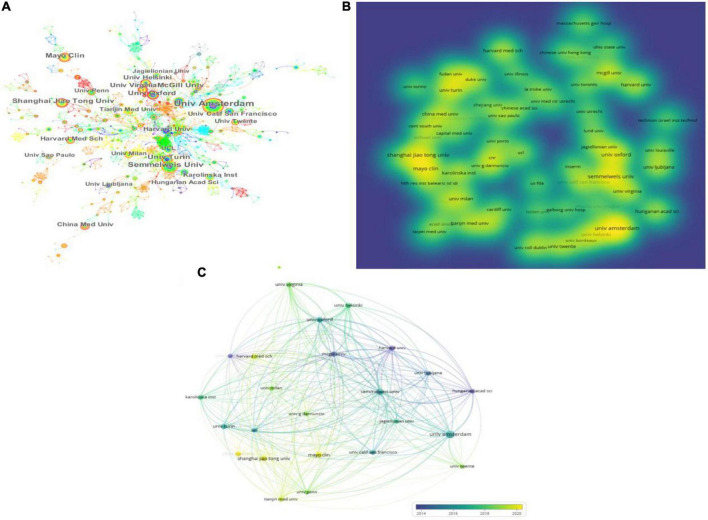
Institution articles on platelet exosomes. **(A)** CiteSpace-based interinstitutional collaboration. **(B)** VOSviewer-based density map of institution distribution. **(C)** VOSviewer-based institutional temporal.

A total of 383 institutions worldwide have contributed to research in this area, with the top 10 institutions listed in Table 3. CiteSpace and VOSviewer were used to create a network visualization of the institutional distribution of global publications. CiteSpace showed 383 nodes and 322 connections, with a network density of 0.0044 (Figure 3A). VOSviewer set a minimum threshold of 7 for the number of documents organized by an institution, and the first 24 institutions met the threshold (Figures 3B,C).

**TABLE 3:** The top 10 institutions with the most publications in the field of platelet exosomes.

No.	Institutions	Publications	Centrality	Citations	Average citation rate
1	Amsterdam University	21 (2.72%)	0.04	1,981	94.31
2	Semmelweis University	15 (1.94%)	0.03	2,297	153.13
3	Oxford University	12 (1.55%)	0.08	1,148	95.67
4	Mayo Clinic	12 (1.55%)	0.02	219	18.25
5	Shanghai Jiao Tong University	12 (1.55%)	0.03	535	44.58
6	McGill University	11 (1.42%)	0.04	1,711	155.55
7	Turin University	11 (1.42%)	0.07	835	75.91
8	Helsinki University	10 (1.30%)	0.01	572	57.20
9	Virginia University	10 (1.30%)	0.01	141	14.10
10	Harvard University	9 (1.17%)	0.08	1,247	138.56

Looking at the global distribution of research publications on exosomes in platelets, Amsterdam University (21, 2.72%) and Semmelweis University (15, 1.94%) topped the list. Several institutions, including Oxford University (0.08), Harvard University (0.08), and Turin University (0.07), showed high centrality among themselves, appearing as purple circles in Figure 3A, indicating that these institutions may have played a key role in the study of exosomes in platelets. Then, density visualization was used to show the institutional release centers where publications were published (Figure 3B); overlay visualization could represent the year of concentration of individual institutional publications (Figure 3C). In terms of total citations, the top three institutions were Semmelweis University (2,297 citations), Amsterdam University (1,981 citations), and McGill University (1,711 citations). Semmelweis University (6,323), Oxford University (5,302), and Amsterdam University (4,567) were the most prominent in terms of total link strength, indicating the above institutions’ dominance in the field. Furthermore, publications from Amsterdam University, Semmelweis University, Oxford University, and Harvard University were primarily concentrated in 2014–2018; publications from Shanghai Jiao Tong University, Fudan University, the Mayo Clinic, and China Medical University were primarily concentrated in 2018–2022; and articles from China Medical University were primarily published after 2020. Oxford University, Harvard University, and Turin University were actively collaborating.

### Journal distribution analysis

**FIGURE 4:**
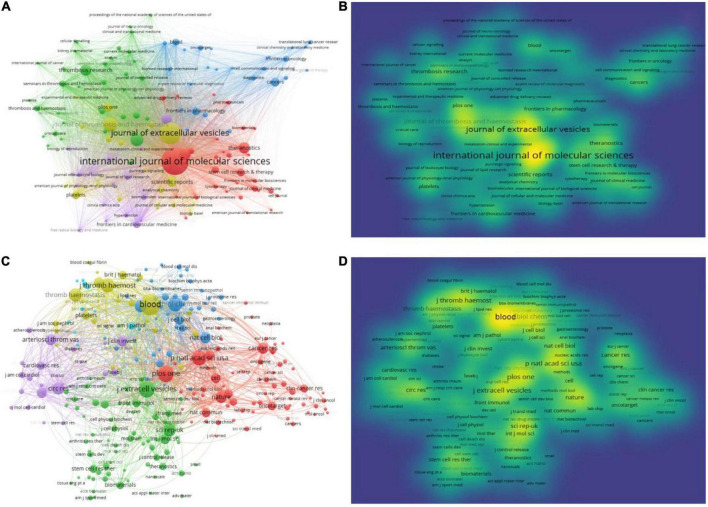
Journals that published articles on platelet exosomes. **(A)** VOSviewer-based journal collaboration. **(B)** VOSviewer-based density map of journals’ distribution. **(C)** VOSviewer-based collaboration among co-cited journals. **(D)** VOSviewer-based density map of article distribution among co-cited journals.

A comprehensive visual analysis of the journals revealed the core journals on exosomes in platelet research. VOSviewer set the minimum number of publications from one to two. A total of 396 journals were used to visualize the network and density of journals publishing studies on exosomes in platelets, with the top 129 journals reaching the threshold (Figures 4A,B).

The *International Journal of Molecular Sciences* (IF: 6.208, 2021) published the most articles, with 37, followed by the *Journal of Extracellular Vesicles* (IF: 17.337, 2021), with 27. The *Journal of Thrombosis and Hemostasis* (IF: 16.036, 2021) had 15 publications, and *Frontiers in Immunology* (IF: 8.786, 2021) had 14 publications. *Blood* had the highest IF (25.476, 2021), followed by the *Journal of Extracellular Vesicles* with an IF of 17.337. The analysis of the source distribution of published articles aided in identifying core journals. Subsequently, the Eigenfactor Score (EF), SCImago Journal Rank (SJR) 2021, and H-index of the top 20 most published journals were searched, and it was found that *PLOS One* had the highest EF (EF: 1.814); *Journal of Extracellular Vesicles* had the highest SJR with SJR 4.878; and Blood had the highest H-index value (H-index: 484), as shown in Table 4.

**TABLE 4:** Impact index of the top 20 journals with the largest number of articles.

No.	Journal	Publications	Impact factor (IF) 2021	Eigenfactor score (EF)	SCImago journal rank (SJR) 2021	H-index
1	International Journal of Molecular Sciences	37 (4.79%)	6.208	0.055	1.176	195
2	Journal of Extracellular Vesicles	27 (3.50%)	17.337	No records	4.878	84
3	Journal of Thrombosis and Hemostasis	15 (1.94%)	16.036	0.042	3.815	191
4	Frontiers in Immunology	14 (1.81%)	8.786	0.040	2.331	155
5	Cells	11 (1.43%)	7.666	No records	1.452	66
6	Theranostics	11 (1.43%)	11.600	0.010	2.061	115
7	Thrombosis Research	11 (1.43%)	10.407	0.020	1.678	118
8	Frontiers in Cell and Developmental Biology	10 (1.30%)	6.081	No records	1.440	67
9	Scientific Reports	9 (1.17%)	4.996	0.209	1.005	242
10	Cancers	8 (1.04%)	6.575	No records	1.349	92
11	Plos One	8 (1.04%)	3.752	1.814	0.852	367
12	Frontiers in Pharmacology	7 (0.91%)	5.988	0.013	1.143	104
13	Platelets	7 (0.91%)	4.236	0.005	0.869	72
14	Stem Cell Research Therapy	7 (0.91%)	8.079	No records	1.330	90
15	Blood	6 (0.78%)	25.476	0.351	4.834	484
16	Frontiers in Cardiovascular Medicine	6 (0.78%)	5.846	No records	1.443	44
17	Frontiers in Physiology	6 (0.78%)	4.755	0.026	1.126	122
18	International Journal of Nanomedicine	6 (0.78%)	7.033	0.025	1.032	145
19	Cellular and Molecular Life Sciences	5 (0.65%)	9.207	0.044	2.424	233
20	Cytometry Part A	5 (0.65%)	4.714	No records	1.469	95

The analysis of co-cited journals was then performed to plot the network visualization and density (Figures 4C,D) to understand the hotness of cited journals and the correlation between journals. *Blood* (IF: 25.476, 2021), *PLOS One* (IF: 3.752, 2021), *Proc Natl Acad Sci USA* (IF: 12.779, 2021), and the *Journal of Extracellular Vesicles* (IF: 17.337, 2021) were found to be the hottest publication centers, with the most prominent impact of studies on exosomes in platelets.

**FIGURE 5:**
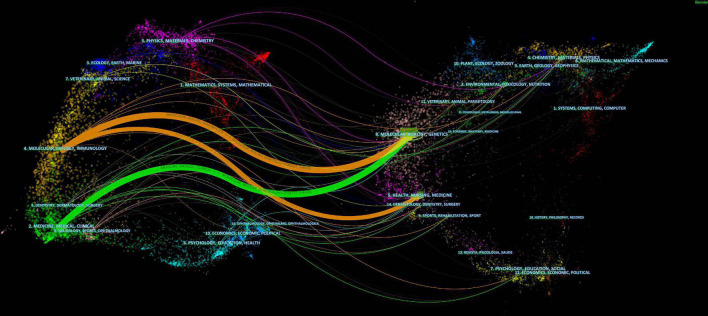
CiteSpace-based dual-map overlay of journals with exosomes in platelets.

The colored paths between the dual-map overlay of the journals represent the distribution of the relationship between citing journals (left) and cited journals (right) (Figure 5). There are currently three primary citation pathways in the global literature related to exosomes in platelets.

Two of the orange pathways show studies from health/nursing/medical journals and molecular/biology/immunology journals that were most likely cited by molecular/biology/genetics journals. The green pathway shows studies from medicine/medical/clinical journals that were mostly cited by molecular/biology/genetics journals.

### Author distribution analysis

**FIGURE 6:**
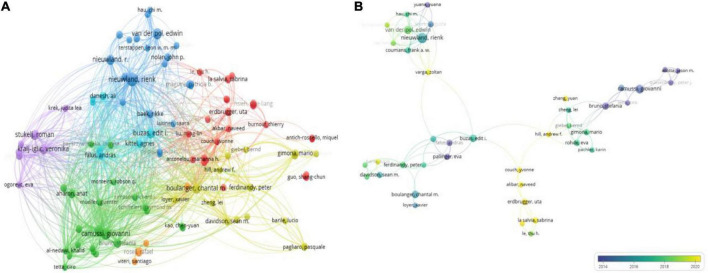
Visualization map of authors involved in platelet exosome research publications. **(A)** Author collaboration based on VOSviewer. **(B)** Collaboration between co-cited authors using VOSviewer.

From 2000 to 2022, 4,598 authors worldwide were involved in exosome-related studies on platelets, and Table 5 shows the authors who contributed the most prominent number of articles and citations. VOSviewer set a minimum number of citations threshold of three, and 108 authors met the threshold for network visualization analysis (Figure 6A). Nieuwland Rienk (17) and Van Der Pol Edwin (11) from Amsterdam University in the Netherlands had the most publications, followed by Camussi from Torino University in Italy, with 8 publications, and Rak Janusz from McGill University in Canada, who had 7.

**TABLE 5:** The top authors in the field of platelet exosomes, ranked by publication and citation numbers.

	Authors	Country	Affiliation	Publications	Citations
Top publications (*n* ≥ 6)	Nieuwland Rienk	Netherlands	Amsterdam University	17	1,879
	Van Der Pol Edwin	Netherlands	Amsterdam University	11	1,274
	Camussi Giovanni	Italy	Torino University	8	1,804
	Rak Janusz	Canada	McGill University	7	1,553
	Boulanger Chantal M	France	Université Paris Descartes	6	826
	Buzas Edit I	Hungary	Semmelweis University	6	1,569
	Kralj-lglic veronika	Slovenia	Ljubljana University	6	141
	Stukelj Roman	Slovenia	Ljubljana University	6	82
	Zhang Wei	China	Tianjin Medical University	6	131
Top citations (n ≥ 1,000)	Bruno Stefania	Italy	Torino University	5	1,903
	Nieuwland Rienk	Netherlands	Amsterdam University	17	1,879
	Camussi Giovanni	Italy	Torino University	8	1,804
	Falus Andras	Hungary	Semmelweis University	4	1,699
	Pallinger Eva	Hungary	Semmelweis University	4	1,699
	Kittel Agnes	Hungary	Hungarian Academy of Sciences	4	1,666
	Simpson Richard J.	Australia	La Trobe University	3	1,654
	Buzas Edit I.	Hungary	Semmelweis University	6	1,569
	Rak Janusz	Canada	McGill University	7	1,553
	Cantaluppi Vincenzo	Italy	Piemonte Orientale University	4	1,297
	Al-Nedawi Khalid	Canada	McGill University	3	1,159
	Meehan Brian	Canada	McGill University	3	1,159

The number of citations for a publication highlights the author’s academic influence, and Table 5 lists the top 12 authors. Bruno Stefania from Torino University in Italy has published only 5 articles with 1,903 citations, indicating that he has made significant contributions to the field of exosomes in platelets. We then used VOSviewer to analyze the visualization of the shortlisted publications’ co-cited author network (Figure 6B). Nodes represent authors; the node size reflects the number of publications; the lines represent author collaboration; and the thickness represents the tightness of the collaboration. It is intuitively clear that Nieuwland Rienk, Buzas Edit I, Camussi Giovanni, and Erdbrugger Uta form the main communication and collaboration network in this research area. When combined with overlay visualization, it is clear that the collaborative network team led by Erdbrugger Uta has the most recent research publications.

### Co-cited references and references burst

**FIGURE 7:**
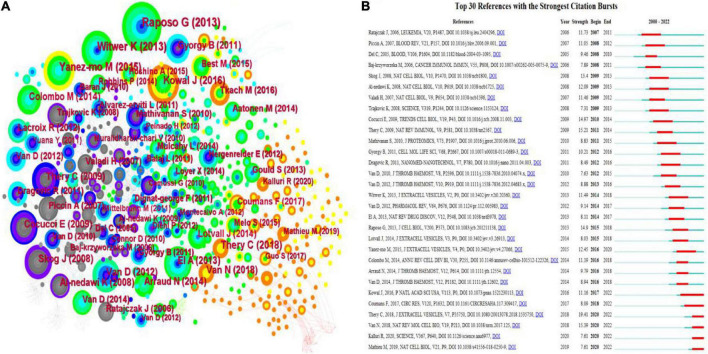
A visualization map of co-cited references in platelet exosome research. **(A)** CiteSpace-based collaboration between co-cited references. **(B)** The top 30 references with the strongest citation bursts are based on CiteSpace.

Co-cited references refer to the third cited article in a reference list of two references, so their simultaneous appearance forms a co-citation pattern. CiteSpace’s co-cited references visualization shows 912 nodes and 2,069 connections with a network density of 0.005 (Figure 7A). Table 6 lists the top 10 most frequently cited references. The first three references were cited more than 80 times. The most highly regarded reference topic was “Extracellular vesicles: exosomes, microvesicles, and friends,” a review that explains how platelets release exosomes in response to thrombin receptor activation after stress.

**TABLE 6:** The top 10 references in the field of exosome that have received the most citations.

No.	Title	Corresponding author	Journal	IF	Publication year	Total citations	Level of evidence
1	Extracellular vesicles: exosomes, microvesicles, and friends	Raposo Graca	Journal of Cell Biology	8.077	2013	129	Review
2	Standardization of sample collection, isolation and analysis methods in extracellular vesicle research	Witwer Kenneth W.	Journal of Extracellular Vesicles	17.337	2013	93	*In vitro* human
3	Biological properties of extracellular vesicles and their physiological functions	Yanez-Mo Maria	Journal of Extracellular Vesicles	17.337	2015	80	Review
4	Proteomic comparison defines novel markers to characterize heterogeneous populations of extracellular vesicle subtypes	Kowal Joanna	Proceedings of the National Academy of Sciences of The United States of America	12.779	2016	79	*In vitro* human
5	Minimal information for studies of extracellular vesicles 2018 (MISEV2018): a position statement of the International Society for Extracellular Vesicles and update of the MISEV2014 guidelines	Thery Clotilde	Journal of Extracellular Vesicles	17.337	2018	77	*In vitro* human animal
6	Membrane vesicles as conveyors of immune responses	Thery Clotilde	Nature Reviews Immunology	108.555	2009	76	Review
7	Shedding microvesicles: artefacts no more	Cocucci E	Trends in Cell Biology	21.167	2009	76	Review
8	Classification, functions, and clinical relevance of extracellular vesicles	Van der Pol Edwin	Pharmacological Reviews	18.923	2012	71	Review
9	Glioblastoma microvesicles transport RNA and proteins that promote tumor growth and provide diagnostic biomarkers	Skog Johan	Nature Cell Biology	28.213	2008	69	*In vitro* human
10	Shedding light on the cell biology of extracellular vesicles	Van Niel Guillaume	Nature Reviews Molecular Cell Biology	113.915	2018	60	Review

The top 30 references for platelet exosome-related studies were visualized for the number of citation bursts and are shown in Figure 7B. The first citation explosion of references appeared as early as 2005, during which the references of citation bursts were constantly updated, indicating that the global scholars’ research fervor for exosomes in platelets remains hot and worthy of in-depth exploration. Notably, Kowal et al.’s (22) article has sparked considerable interest.

### The analysis of hotspots and the frontiers

**FIGURE 8:**
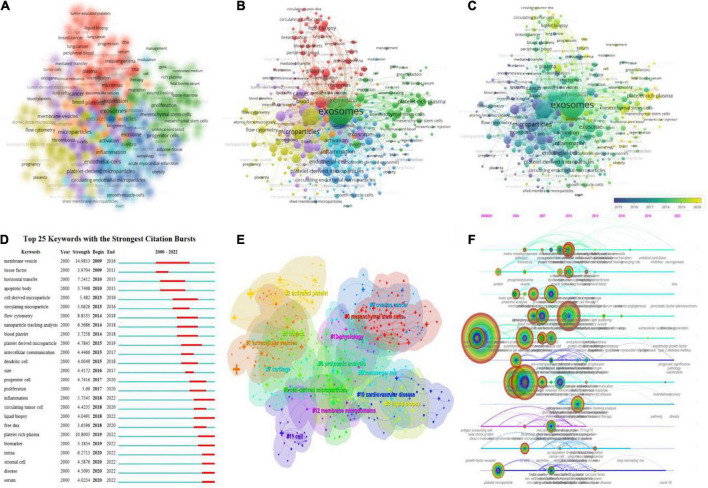
Keyword mapping in platelet exosome studies. **(A)** VOSviewer-based keyword density visualization. **(B)** VOSviewer-based keyword network visualization. **(C)** VOSviewer-based keyword chronological visualization. **(D)** CiteSpace’s top 25 keywords with the strongest citation bursts. **(E)** CiteSpace-based keyword clustering analysis. **(F)** CiteSpace-based keyword timeline visualization.

Keyword network visualization analysis aids in the advancement of exosome research in platelets and their intrinsic connections. Table 7 shows the top 20 high-frequency keywords. VOSviewer was used to set a minimum threshold of five keyword occurrences, and 307 keywords were shown (Figures 8A,B).

**TABLE 7:** The top 20 keywords for platelet exosomes.

No.	Keywords	Count	Centrality	No.	Keywords	Count	Centrality
1	Exosome	571	0.03	11	Biomarker	82	0.03
2	Extracellular vesicle	310	0.06	12	Inflammation	75	0.05
3	Microvesicle	247	0.08	13	Mesenchymal stem cell	74	0.01
4	Microparticle	152	0.01	14	Endothelial cell	74	0.09
5	Platelet	129	0.02	15	Platelet microparticle	73	0.01
6	Activated platelet	99	0.05	16	Cancer	70	0.01
7	MicroRNA	87	0.00	17	Platelet-derived microparticle	66	0.03
8	Cell	87	0.07	18	Membrane vesicle	64	0.00
9	Tissue factor	84	0.01	19	Plasma	61	0.04
10	Expression	83	0.07	20	Stem cell	57	0.06

In the overlay visualization, the keywords are colored differently based on their annual distribution (Figure 8C). For example, “microparticles,” “platelet microparticles,” and “activated platelets” primarily appeared in 2015, while “exosomes,” “inflammation,” and “plasma” primarily appeared in 2018. The keywords “exosomes,” “inflammation,” and “plasma” primarily appeared in 2018, while the keywords “platelet-rich plasma,” “DNA,” and “mesenchymal stem cells” primarily appeared in recent years. Keyword emergence in recent years indicates that related fields are becoming more popular and are likely to become more popular in the future. Figure 8D displays the top 25 keywords with the strongest citation burst (minimum duration threshold set to 1). The most frequently used keywords in the last 3 years were “serum disease,” “stromal cell,” “miRNA,” “biomarker,” “platelet-rich plasma,” “inflammation,” and “liquid biopsy.” In addition, CiteSpace was used to perform keyword clustering analysis (Figure 8E), with circles and labels forming 14 clusters with colors identifying which cluster it belongs to, such as #0 mesenchymal stem cells, #1 extracellular vesicles, #2 activated platelets, #3 liquid biopsy, and others. The time axis clustering (Figure 8F) revealed that “mass spectrometry,” “flow cytometry,” “lipoprotein,” “NAD(P)H oxidase,” “liquid droplets,” “microparticles,” “RNA,” “COVID-19,” and others are the focus of current research in this field and can guide the future direction.

## Discussion

### General information

Undoubtedly, knowledge management is one of the most important concerns for scientists in the information age. According to a search of the WoS database as of 2 August 2022, 4,598 authors from 383 institutions in 69 countries/regions had published 722 studies on platelet exosomes in 396 academic journals.

The number of publications in a research field reflects productivity, and the number of citations demonstrates research impact, which are the two main bibliometric indicators for assessing the level of academic research. There was no research in this field between 2000 and 2006.

Between 2007 and 2013, a small number of articles appeared in the early stages of platelet exosome-related research. From 2014 to 2017, the number of global publications began to increase, and more researchers began to focus on the role of platelet exosomes (23–25). The number of published articles on platelet exosome research has increased over the last 5 years, indicating that the field is expanding and will continue to attract attention.

A comprehensive analysis of visualization tools reveals that the USA and the PRC have the highest productivity in studying platelet exosomes. Amsterdam University in the Netherlands and Semmelweis University in Hungary are 2 of the top 10 research institutions in terms of publications. Although countries have collaborative networks, as shown in Figure 2B, the breadth and intensity of collaboration are not ideal, particularly between the two major publication-contributing countries, the USA and the PRC, where there is essentially no collaboration in platelet exosome-related research. In contrast, collaborations among the USA, Germany, Poland, and other countries are relatively more common. Some research institutions, such as Oxford University, Harvard University, and Turin University, have strong collaborative relationships. Despite publishing the most papers, we discovered that Amsterdam University and Semmelweis University collaborate very little. Overall, most collaborating institutions are limited to internal contacts with little cross-country collaboration and exchange of results, which impedes the long-term development of this research area. Therefore, it is strongly recommended that the PRC and the USA remove academic barriers with research institutions in other countries and work to improve scientific cooperation and exchange in the field of exosomes in platelets.

Most studies on exosomes in platelets published in the *International Journal of Molecular Sciences* (IF = 6.208, 2021) deserve to be highlighted. *Blood* (IF = 25.476, 2021) has the highest IF among the top 20 journals in terms of the number of publications, followed by the *Journal of Extracellular Vesicles* (IF = 17.337, 2021). Regarding co-cited journals, *Blood* (IF = 9.461, 2021), *PLOS One* (IF = 3.24, 2021), *Proc Natl Acad Sci USA* (IF = 6.277, 2021), and the *Journal of Extracellular Vesicles* (IF = 6.277, 2021) are the hottest publishing centers, which are most closely associated with other journals and provide strong academic support for the study of exosomes in platelets. Most relevant research results in this field are currently published in molecular, biological, and immunological journals, with most publications focusing on basic medical research. Therefore, in the future, we should actively translate research findings into clinical practice and promote practical applications in related disciplines.

Highlighting influential researchers, such as coauthors or co-cited authors in a given field, can provide additional research directions and academic guidelines for scholars in the same field (26). Nieuwland Rienk and Van Der Pol Edwin, both from Amsterdam University in the Netherlands, are the top two most productive authors in terms of author contributions and co-cited authors, with 17 and 11 articles, respectively, with Nieuwland Rienk’s article also ranking second in the number of citations in the co-citation network, indicating that this author has made an outstanding contribution to exosomes in platelet research. Furthermore, the map of authors and co-cited authors identifies potential collaborators and influential research groups (27). Nieuwland Rienk’s team investigated the isolation of blood-derived extracellular vesicles (28) and their purification as disease biomarkers (29), taking into account the challenges of platelet activation and aggregation during isolation. It was proposed that centrifugation conditions meet the prerequisites for reducing or preventing platelet activation using low pH compounds that inhibit platelet activation, minimal centrifugal force, the simplest separation steps, standardizing the method of isolating platelet-derived extracellular vesicles (EVs), and assessing the purity of biomarkers after centrifugation using the Stokes equation. Nieuwland Aatonen et al. (28) also stated that it is becoming more common to use EVs like “particles” and “exosomes” for clinical diagnosis and treatment. This means that bulk tests for EVs of any origin, including PLT-Exos, are widely used. In addition to immunoblotting and enzyme-linked immunosorbent assay (ELISA), the most sensitive assays are micronuclear magnetic resonance, surface plasmon resonance, and time-resolved fluorescence immunoassays, which can detect clinical samples and accurately identify disease biomarkers down to a 10,000-fold EV concentration.

### Knowledge base

Co-cited references are those cited by multiple other publications and can be used as the foundation for research in a particular field (30). The top 10 co-cited references were investigated regarding extracellular vesicle collection, isolation, analysis methods, clinical applications as biomarkers, and physiological functions performed. Raposo Graca et al. published the most frequently cited article in the *Journal of Cell Biology* in 2013 (31) (citation = 291), describing the steps for isolating extracellular vesicles and the methods for improving and standardizing analysis methods. Using EVs as biomarkers in clinical settings, vaccinations, or drug delivery devices needs more precise and standardized purification techniques. Witwer Kenneth W et al. published an article in 2013 in the *Journal of Extracellular Vesicles* (32) (citation = 93), based on the International Society for Extracellular Vesicles (ISEV) Special Symposium held in New York City in October 2012, to provide a comprehensive review of current knowledge about many gaps in technology related to EVs. It describes the isolation and analysis of EVs and the purification and analysis of relevant RNA molecules.

The increase in citations may reflect the dynamic changes and hotspots of related research. The most explosive reference is the guide published in 2018 by Clotilde Théry’s research group in the *Journal of Extracellular Vesicles* (33) (strength = 19.41), based on ISEV’s proposed Minimal Information for Studies of Extracellular Vesicles (“MISEV”) guidelines for the field in 2014, revised and updated the 2014 version of the MISEV recommendations, providing insight into the findings and developments in the EV field and providing recommendations for the clinical application of protein markers. Second, a study published in *Nature Reviews Molecular Cell Biology* in 2018 (34) (strength = 15.39) by Guillaume van Niel et al. points to EV as a new form of intercellular communication that allows cells to exchange proteins, lipids, and genetic material that carry specific protein, lipid, or RNA species, which then determine their fate and function. EV interactions with recipient cells can have various effects on target cells, ranging from stimulating signaling pathways to providing nutritional support, depending on the mode of interaction and the intracellular fate of the vesicles. Thery C et al. published *Nature Reviews Immunology* in 2009 (35) (strength = 15.21), focusing on the role of membrane vesicles, particularly exosomes, in communication between the immune cells and between tumors and immune cells. It is worth noting that secreted vesicles can change the genetic characteristics of recipient cells. Exosomes from dendritic cells or tumors from cancer patients have also been used for immunotherapy with limited clinical response.

Recent preclinical studies on exosomes will help future clinical trials, which should use both exosomes and other ways to change the immune system.

### The hotspot and trending

Acquiring and tracking the latest research advances have always been scholars’ most important scientific skill. In particular, rkeyword co-occurence and clustering analysis in bibliometrics can highlight academic field frontiers (36); keyword bursts and timeline views can present the evolution and future development of new hotspots (37); and clustering of co-cited literature and citation bursts can reveal emerging themes in the subject area (38). Keyword co-occurrence, clustering, bursts, timeline views, and citation bursts of co-cited references were used to summarize the following three research hotspots and trends in this study (Tables 6, 7 and Figures 7, 8).

#### Methodological considerations for PLT-Exos isolation and purification

Compared to other blood components, platelets secrete the most exosomes and the most abundant content species (39, 40). For the isolation and purification of PLT-Exos, in addition to the differential centrifugation method, traditional extraction methods include filter centrifugation, density gradient centrifugation, immunomagnetic bead method, phosphatidylserine affinity method, chromatography or special exosome isolation kits for extraction (41). Alternatively, as assays are updated and upgraded, researchers can also use flow sorting using CD9, CD63, and CD81 specific markers (42), direct observation calculations using single-cell exosome electron projection fluorescence microscopy (43), asymmetric flow field-flow grading method to identify nanoscale particles and vesicles (44), and others.

The mechanism of exosome regulation during thrombosis is complex and diverse and is closely related to various cells and cellular products (45). The study of exosomes also plays a critical role in investigating the pathological mechanisms of thrombosis and searching for better therapeutic approaches. Nieuwland Rienk’s team worked on the analysis of the isolation of blood-derived EV (28) and its purification as a biomarker (29). They standardized the method of isolating platelet-derived EVs using low pH compounds that inhibit platelet activation, minimal centrifugal force, and the most simplified separation steps, and a model based on the Stokes equation to assess the purity of biomarkers after centrifugation.

In addition, Nieuwland Rienk et al. stated that there is acceptance of the use of EVs, including platelet-derived ones, for clinical diagnosis and treatment. It should be noted that, in addition to immunoblotting and ELISA, the two main native immunoassays, new assays such as micronuclear magnetic resonance, surface plasmon resonance, and time-resolved fluorescence immunoassays are more sensitive and can be used down to 10,000 times the EV concentration, providing a significant boost to the detection of clinical samples and the precise determination of disease biomarkers.

Based on this, future considerations for PLT-Exos isolation and purification should focus on reducing experimental influencing factors and enhancing the sensitivity of diagnostic markers applied to clinical diseases. On the one hand, it is necessary to optimize the stability of platelet storage *in vitro* and explore the reduction of PLT-Exos isolation steps in blood samples; on the other hand, it is necessary to actively develop new isolation and extraction processes and to improve the purification concentration, sensitivity, and accuracy by combining micro-magnetic resonance and fluorescence immunoassay methods, which will be a popular trend in future PLT-Exos isolation and purification methods. Purification methods will be a popular trend in the future.

#### Function and mechanism of PLT-Exos

Platelets form the thrombus and inhibit its formation by secreting exosomes. In recent studies, exosomes have been linked to thrombosis, and exosomes produced by various cells regulate thrombosis (46, 47). By mediating platelet aggregation and coagulation pathways, PLT-Exos miRNAs can enhance the delivery of atherosclerotic material to injured endothelial cells and macrophages, potentially accelerating the development of vascular plaques, thrombosis, and atherosclerosis (48). Furthermore, PLT-Exos inhibit platelet aggregation *in vitro* and reduce platelet adhesion to exposed collagen in damaged vessels induced by high shear *in vivo*. Exosomes inhibit vaso-occlusive thrombosis in an iron trichloride-induced arterial injury model by reducing platelet reactivity (49). PLT-Exos have been shown to play a role in cellular communication during inflammation and the immune response. First, PLT-Exos act as a source of pro-inflammatory cytokines, which enhance local and systemic inflammatory responses and stimulate their production in various cells. Second, PLT-Exos induce granulocyte migration to inflamed tissues and facilitate their delivery to endothelial cells and macrophages at the site of vascular lesions. In a thrombin-stimulated vascular endothelial cell inflammation-thrombosis model, PLT-Exos regulate the thrombotic–inflammatory response and inhibit the inflammatory response (50). Moreover, platelet procoagulant activity is linked to the release of platelet extracellular bodies.

Platelets secrete the vast majority of exosomes in platelet-rich plasma (PRP). Exosomes are stored in platelet α-granules and multivesicular bodies and then secreted extracellularly in response to certain stimuli to participate in relevant physiological processes. Torreggiani et al. (51) found that bone marrow mesenchymal stem cells (BMMSCs) could take up fluorescently labeled PRP-Exos and that PRP-Exos significantly promoted the proliferation of human BMMSCs with increasing concentrations of PRP-Exos. Following in the footsteps of Torreggiani et al. (51), who isolated exosomes from PRP and demonstrated that PRP-Exos promote tissue regeneration, Tao et al. (52) elucidated that PRP-Exos effectively promote epithelial regeneration in chronic skin wounds by activating Yes-associated protein in a diabetic rat model, suggesting multiple miRNAs associated with PRP-Exos play an important role in angiogenesis. Sun et al. (53) found that PRP-Exos in patients with acute coronary syndrome contained high levels of miR-126 and angiogenic factors, which promoted the proliferation and migration of human umbilical vein endothelial cells, regulating angiogenesis.

It is reasonable to believe that the central position of platelet-rich plasma-derived exosomes (PRP-Exos) in PLT-Exos-related studies will continue to take advantage of its strong potential in the future, focusing on pro-thrombotic, anti-inflammatory, anti-apoptotic, pro-angiogenic, and proliferative functions *via* the transport of miRNAs and proteins.

#### Clinical application of PRP-Exos as a biomarker

Exosomes are found in peripheral blood and contain various markers. The current biological coagulation cascade has been identified, but the various molecular mechanisms of coagulation remain unknown. The clinic currently uses D-dimer as a predictor of thromboembolic disease, but it has poor specificity (54). Wen et al. (55) found that stimulating human umbilical vein endothelial cells with circRNA-0006896-rich exosomes isolated from the sera of patients with unstable angina/vulnerable plaque atherosclerosis increased miRNA-1264 and DNMT1 protein expression, activated the JNK/STAT3 signaling pathway, and promoted vascular endothelial cell proliferation and migration. Exosomes containing miR-208a in the serum promote thrombosis in acute coronary syndrome (56). Platelets aggregate and adhere to local microstenoses when endothelial cells are damaged, accumulating components such as thrombin and fibrinogen at the damaged site. PRP-Exos miRNA can cause or delay the development of atherosclerosis by inducing or suppressing the inflammatory response, making it a biomarker for predicting thrombosis and a therapeutic target (57). Li et al. (58) found that BMMSCs secreted paracrine miR-451-5p-rich exosomes, which promoted the massive secretion of PAI-1 from vascular endothelial and vascular smooth muscle cells, resulting in abnormal fibrinolysis in the microcirculation and thrombosis.

Exosomes can be used as a therapeutic tool. Hou et al. (59) used electrostatic binding to immobilize exosomes loaded with natural factors on a polydopamine-coated material and found that they inhibited platelet surface endothelial cell adhesion molecule-1, CD31 expression, reduced pro-inflammatory macrophages (M1 type) attached to vascular endothelial cells, and prevented late thrombosis and restenosis after stent implantation. It has been demonstrated that EV acts as a tumorigenesis regulator by transferring its cargo molecules (miRNA, DNA, proteins, cytokines, receptors, and others) between cancer cells and cells in the tumor microenvironment (14). Platelet EVs (PEVs) form during platelet activation or apoptosis. PEVs have the most diverse EV population and are the most abundant EV population in the circulatory system. Because the role of platelets in cancer development is well understood, PEV crosstalk can promote proliferation, alter the tumor microenvironment, and favor metastasis. PEVs and platelets have many common functional properties. With PEV drug loading, there could be a new biocompatible and non-immunogenic next-generation natural vector-targeted drug (60). In a study, Kailashiya et al. documented the uptake of adriamycin-loaded PEV (doxo-PEV) from leukemia cell lines (HL60 and K562 cells) and primary cells harvested from the whole blood of newly diagnosed leukemia patients. A higher percentage of doxo-PEV was transferred to leukemia cells than free adriamycin, which could be used to improve therapeutic efficacy and reduce drug adverse effects (61).

It was anticipated that PRP-Exos act as a key functional effector linking inflammation, hypercoagulability, angiogenesis, endothelial cell dysfunction, and apoptosis, affecting the onset, progression, and thrombosis of vascular thrombotic diseases. The future focus will be on PRP-Exos miRNAs, mesenchymal stem cells, and platelet microparticles to further explore minimally invasive biomarkers for predicting, diagnosing, and monitoring the pathological state of thrombotic diseases, as well as new specific drug targets for the treatment of thrombotic disease formation.

In summary, PRP-Exos are involved in pathophysiological processes such as inflammation, thrombosis, cardiovascular and cerebrovascular diseases, autoimmune diseases, and hematological malignancies. They have emerged as potential biomarkers or prognostic factors for many clinical diseases. On the one hand, because platelets are prone to activation and aggregation and exosomes are small in size and number, with different physicochemical properties and the complexity of the surrounding fluid, the separation and purification of their exosomes remain a challenging area that requires further optimization and standardization. On the other hand, the diagnostic and therapeutic potential of PRP-Exos for clinical diseases remains an open question, and a significant challenge for the development of exosomes for clinical applications is the development of sterile, scalable, reproducible, and efficient methods of clinical exosome production. Therefore, exploring the potential mechanisms of exosomes in platelets and developing platelet-derived exosomes are important research avenues for addressing the diagnosis and prediction of multisystem clinical diseases.

### Advantages and shortcomings

Overall, the strengths of this study are clear. For the first time, we used a bibliometric system to analyze studies of exosomes in platelets, which can provide academic direction for scholars interested in related research. Second, we used two bibliometric tools that have been authoritatively recognized in the field of bibliometrics: VOSviewer and CiteSpace. We combined their strengths to conduct a research survey in this field, making our analysis process as comprehensive as possible. Finally, bibliometric analysis allows for a more complete and comprehensive exploration of the research hotspots and future academic directions of platelets in COVID-19 than traditional reviews.

Of course, several parts still need to be improved and enhanced. First, the academic data in this research area are all from the WoS database, which may result in the absence of some relevant research literature; second, we uniformly used English as the only language for searching the literature, and there is a possibility of filtering some quality non-English literature data; and finally, CiteSpace and VOSviewer have no control over the quality of the collected literature data, which may undermine the credibility of the data visualization. Therefore, a more comprehensive and accurate visual analysis of the literature in this field is required. However, a visualization-based literature analysis is unquestionably important for focusing on the research hotspots and trends of exosomes in platelets.

## Conclusion

Exosomes play an important role in platelet research, both in basic research and clinical exploration. There is no doubt that the study of PLT-Exos has piqued the interest of scholars all over the world. Visual analysis of exosomes in platelets using CiteSpace and VOSviewer software revealed significant trends. The explosive growth in the number of publications in international core journals indicates the significant impact of research in this field, with the USA and the PRC leading the way. However, cooperation and communication between countries and institutions must be improved. All researchers should try to improve their articles’ impact while publishing more research. Current research in this area must actively integrate clinical problems with basic research, emphasizing the clinical translation of PLT-Exos mechanistic studies. Exosomes in platelet research are currently focused on “thrombosis,” “anti-inflammatory,” “anti-apoptosis,” “angiogenesis,” “microparticles,” “miRNA,” “stem cells,” and “biomarkers,” which will serve as hot and cutting-edge directions in this academic field.

## Data availability statement

The original contributions presented in this study are included in the article/supplementary material, further inquiries can be directed to the corresponding authors.

## Author contributions

MZ wrote the manuscript. YZ contributed to the study’s concept. SS and XZ analyzed the data. WC drew the images. LS and JL approved the manuscript. All authors contributed to this article, and the submission of the final version of the manuscript was noted and approved by each author.

## Footnotes

1
https://www.scimagojr.com/

2
http://www.eigenfactor.org/index.php
